# Characterization of Anti-Müllerian Hormone (AMH) Gene in Buffaloes and Goats

**DOI:** 10.3389/fvets.2021.627094

**Published:** 2021-03-08

**Authors:** Devika Gautam, Ashutosh Vats, Prasanna Pal, Avijit Haldar, Sachinandan De

**Affiliations:** ^1^Animal Genomics Lab, Animal Biotechnology Centre, ICAR-National Dairy Research Institute (NDRI), Karnal, India; ^2^Animal Physiology Division, ICAR-National Dairy Research Institute (NDRI), Karnal, India; ^3^ICAR-Agricultural Technology Application Research Institute (ATARI), Indian Council of Agricultural Research, Kolkata, India

**Keywords:** Anti-Müllerian hormone, phylogenetic tree, syntenic analysis, Indian water buffalo, goat, reproduction, fertility

## Abstract

The Anti-Müllerian Hormone (AMH) is a member of the transforming growth factor beta (TGF-β) superfamily, playing a significant role in cell proliferation, differentiation and apoptosis. In females, AMH is secreted throughout their reproductive life span from ovaries, whereas in males it is secreted by gonadal cells at a very early stage of testicular development. AMH is a promising marker of ovarian reserve in women and can be used to measure the female reproductive lifespan. In the present study, we cloned and sequenced the GC rich *AMH* gene from Indian riverine buffalo (*Bubalus bubalis)* and goat (*Capra hircus*). Obtained sequences were compared to the AMH sequences of other mammals, and corresponding amino acid sequences revealed that the caprine and bovine AMH sequences are more closely related to each other than to those of other mammals. Furthermore, we analyzed the chromosomal localization of *AMH* genes in mammalian species to understand potential syntenic relationship. The *AMH* gene is localized between the sequences for the *SF3A* and *JSRP1* genes and maintains this precise location in relation to other nearby genes. The dN/dS ratio of *AMH* gene did not indicate any pressure for either positive or negative selection; thus, the physiological function of the *AMH* gene in the reproduction of these two ruminant species remains very vital. Similar to other mammals, the *AMH* gene may be an important indicator for regulating female reproductive biology function in bovine, cetacean, caprine, and camelidae.

## Introduction

Anti-Müllerian hormone (AMH), also known as Müllerian-Inhibiting Substance (MIS), is a well-studied regulatory molecule impacting on reproductive function, and has specifically studied in male sexual differentiation during early embryonic development. AMH is synthesized by fetal Sertoli cells at the time of testicular differentiation and induces regression of the Mullerian ducts that form the base for the development of the oviducts, uterus and upper part of the vagina in males (1). In the absence of AMH, the Mullerian ducts develop into the oviducts, the uterus and the upper part of the vagina (2). Postnatally, serum AMH concentration increases significantly until puberty, followed by a slow decline throughout the rest of life (3). This decline has been attempted to be modeled by a variety of approaches, with a cubic model resulting in the best fit model that delineates the change of plasma AMH level with the advancement of age in cattle and goats (4–6). The gradual decline of serum AMH concentration runs in parallel with the depletion of the number of growing ovarian follicles (7), making this hormone an ideal prognostic biomarker of the ovarian follicular reserve (8, 9), and thus for fertility and herd longevity in cattle farm (10). In addition, AMH may also be used as a diagnostic marker for ovarian functional disorders in domestic animals. Indeed, AMH levels are increased in women with polycystic ovary syndrome (PCOS) (11, 12); however, this question, with particular reference to anestrus and repeat breeding, has not yet been studied in farm animals.

AMH has a molecular weight of 140 kDa, corresponding to 553–575 amino acids and belonging to the transforming growth factor-beta (TGF-β) superfamily (11). It is a dimeric glycoprotein consisting of two identical subunits linked by sulfide bridges and characterized by the N-terminal dimer (pro-region) and C-terminal dimer (TGF-β domain). The AMH protein is encoded by the *AMH* gene that spans over 2.75 Kbp and contains five exons. The gene is located in chromosome 7 in cattle, horses and goats; chromosome 5 in sheep; chromosome 9 in buffalo; and chromosome 2 in pig (12, 13). In Nelore cattle, the three synonymous mutations (rs527023314, rs722016629, and rs134387246) were found in exon 5 of AMH, which may be associated with early pregnancy occurrence and age at first calving in this cattle breed (14). In ovaries, AMH is produced by the granulosa cells of early growing follicles and is proportional to the antral follicle count, which is a significant determinant of the age-related decline in female fertility due to decrease in ovarian reserve (15). AMH expression reaches its peak level in primordial, primary, and secondary follicles and AMH expression is strongest in granulosa cells of preantral and small antral follicles, whereas it decreases once the dominant follicle is selected and is absent in atretic follicles. This dynamic expression was firstly reported in rabbits (16), and then in the rat (17), human (18), cattle (19), chicken (20), sheep (21), mares (22), buffalo (23), goats (24), and pigs (25). These observations strongly suggest that AMH is a dominant regulator of early follicular growth. AMH hormone levels and antral follicle populations can be used as a marker to genetically improve fertility rates in Nelore cattle and to screen for better oocyte donors (26). The *AMH* gene has not been characterized in many domestic animals, specifically large as well as small ruminant species. In India, riverine buffalo are representative of large domestic ruminant, whereas goats are the major small ruminant, and both play an economically important role. Therefore, the objective of the present study was to clone and sequence the *AMH* gene in Indian riverine buffalo and goat. The deduced amino acid sequence of Indian riverine buffalo and goat AMH as well as sixteen other mammalian AMH amino acid sequences was used to understand the phylogenetic relationships to assess potential changes in reproductive processes.

## Materials and Methods

### Ethics Statement

The study was approved (IAEC/41/14) by the Institutional Animal Ethics Committee (IACE), ICAR-NDRI, Karnal.

### Experimental Design

Ovaries of Indian riverine buffalo (*Bubalus bulalis)* and goat (*Capra hircus*) were used to isolate RNA which was subsequently used to amplify the *AHM* cDNA. The resulting fragments were cloned and sequenced. The deduced AMH sequences were used to construct the phylogenetic tree of the *AMH* gene.

### Sample Collection

The *AMH* gene is expressed only in the ovarian granulosa cells and male fetal testis. Hence, 150 ovaries from 75 Indian riverine buffaloes and 30 ovaries from 15 goats of unknown history, parity and cyclic condition were collected from the slaughterhouse at Gazipur, New Delhi and Municipal Corporation (Karnal, Haryana), respectively. The ovaries were washed with 0.9% normal saline supplemented with 100 μg/ml streptomycin. The samples were brought to the laboratory within three to four hours in RNA*later* (AM7021, Thermo Scientific) at 4^o^C. Small follicles were searched and aspirated with the help of a sterile syringe and a needle. Ovaries with a cyst like growth and damaged ones were not used.

### Total RNA Isolation and cDNA Construction

Small follicles from healthy ovaries of Indian riverine buffaloes and goats were aspirated as described (25, 26), and the pooled fluid containing granulosa cells were centrifuged at 500 × *g* for 5 min followed by washing twice with 1x PBS. RNA was isolated according to the manufactures protocol using TRI reagent (T9424, Sigma Aldrich, Merck, Darmstadt, Germany). The quality and quantity of RNA was checked through 1.5% agarose gel electrophoresis and spectrophotometrically through NanoDrop 2000 (Thermo Scientific, Delaware, USA). First strand cDNA was synthesized from 1 to 2 μg of total RNA using SuperScript® III First-Strand Synthesis System (18080051, Invitrogen, USA) for RT-PCR.

### Amplification and Cloning of *AMH* Coding Sequence

The PCR primers were designed based on bovine AMH sequence available at NCBI, USA (Accession No: M13151.1 and NM_173890.1). The primer pair [Forward: 5'AGGATGCCCGGTCCATCTCTCTCT 3' (24bp) and Reverse: 5' ACCGGCAGCCGCATTCGGTGG 3' (21bp)] was used to amplify the 1728bp *AMH* coding sequence of Indian riverine buffalo and goat *AMH* by polymerase chain reaction (PCR) using cDNA as a template. The PCR reaction was carried out with 25 μl a master mix containing 2 μl of cDNA, 10 pmol each of forward and reverse primers, 1x Taq assay buffer, 200 μM of deoxynucleotide triphosphates (dNTPs), 2.5 μl of GC enhancer and 0.75 units Taq DNA polymerase (Bangalore Genei Pvt. Ltd., Bangalore, India). The PCR reaction was performed in a Veriti Thermal Cycler (Applied Biosystem, Thermo Fisher Scientific, USA) by programming the cycling parameters consisting of an initial denaturation at 95^o^C for 3 min followed by amplification for 35 cycles (each consisting of denaturation at 95^o^C for 30 s, annealing at 55^o^C for 30 s and extension at 72^o^C for 2 min). Final extension of 7 min was at 72°C. The PCR amplified DNA fragments were resolved by electrophoresis on 1.5% agarose gel stained with ethidium bromide in 1x tris acetate EDTA (TAE) buffer and visualized in Gel Documentation system (Gel Doc-XR, BioRad, USA) against 1 Kbp DNA ladder (SM0311, Thermo Scientific™). The band corresponding to 1728 bp was carefully excised and DNA was extracted using a NucleoSpin gel extraction Kit according to manufacturer's instruction (740609.250, Macherey-Nagel, Germany). The amplified Indian riverine buffalo and goat *AMH* gene fragments were cloned into a linearized vector (pTZ57R/T) using InsTAclone PCR cloning kit (K1214, Thermo Scientific, USA), which was subsequently used to transform *E. coli* XL-1Blue chemically competent cells using ampicillin (50 mg/ml) as a selection marker for multiplication of the insert. Six to eight recombinant colonies containing a plasmid with insert were enriched on Luria Bertani (LB) broth. The plasmid DNA was extracted by the alkaline lysis method (27) with slight modifications. The positive clones for *AMH* gene were screened with vector-specific primers and sequenced by sanger dideoxy method through capillary sequencing.

### Sequence Acquisition, Multiple Sequence Alignment, and Phylogenetic Tree Construction

Extracted plasmids containing the appropriate inserts were sequenced using universal sequencing primers [M13 (-20)] Forward and [M13 (-20)] Reverse. The chromatogram was analyzed for read quality assessment and vector contamination using VecScreen (http://www.ncbi.nlm.nih.gov). The multiple sequence alignments were performed using the MAFFT tool (for multiple alignment using fast Fourier transform, version:7) (28). The coding regions were translated through the Expasy translate tool (https://web.expasy.org/translate/). The forward and reverse sequences along with the reference sequences were aligned using MAFFT and the assembled entire coding sequences were submitted to GenBank, NCBI. A phylogenetic tree was generated to establish the molecular evolutionary relationship of AMH amino acid sequences of Indian riverine buffalo and goat and with fourteen other mammalian species. The evolutionary history was inferred using the Neighbor-Joining method and the optimal tree with the sum of branch length = 0.89 is shown. The phylogenetic inference was further validated with the bootstrapping (1000 replicates). The passion correction method was used to compute the evolutionary distances and was in the number of amino acid substitutions per site. All ambiguous positions were removed for each sequence pair (pairwise deletion option). There was a total of 623 positions in the final dataset. Evolutionary analyses were conducted in Molecular Evolutionary Genetics Analysis (MEGA X) (29).

### Selection Pressure Analysis

Selection pressure on protein coding gene of mammalian *AMH* was analyzed in Mega-Datamonkey server based on single likelihood ancestor counting (SLAC) method (30). The selection pressures were calculated by comparing the rate of substitutions at silent sites (dS), which were presumed neutral, to the rate of substitutions at non-silent sites (dN). The dN/dS is measured across the whole protein coding sequence between two divergent species for selection. If the ratio = 1, then the whole coding sequence evolved neutrally, then all nucleotide in a sequence is equally likely to change, when 0 <dN/dS<1, it would be under constraint > 1, it would be under positive selection.

## Results

### Amplification of High GC Containing *AMH* Coding Sequence

**Figure 1:**
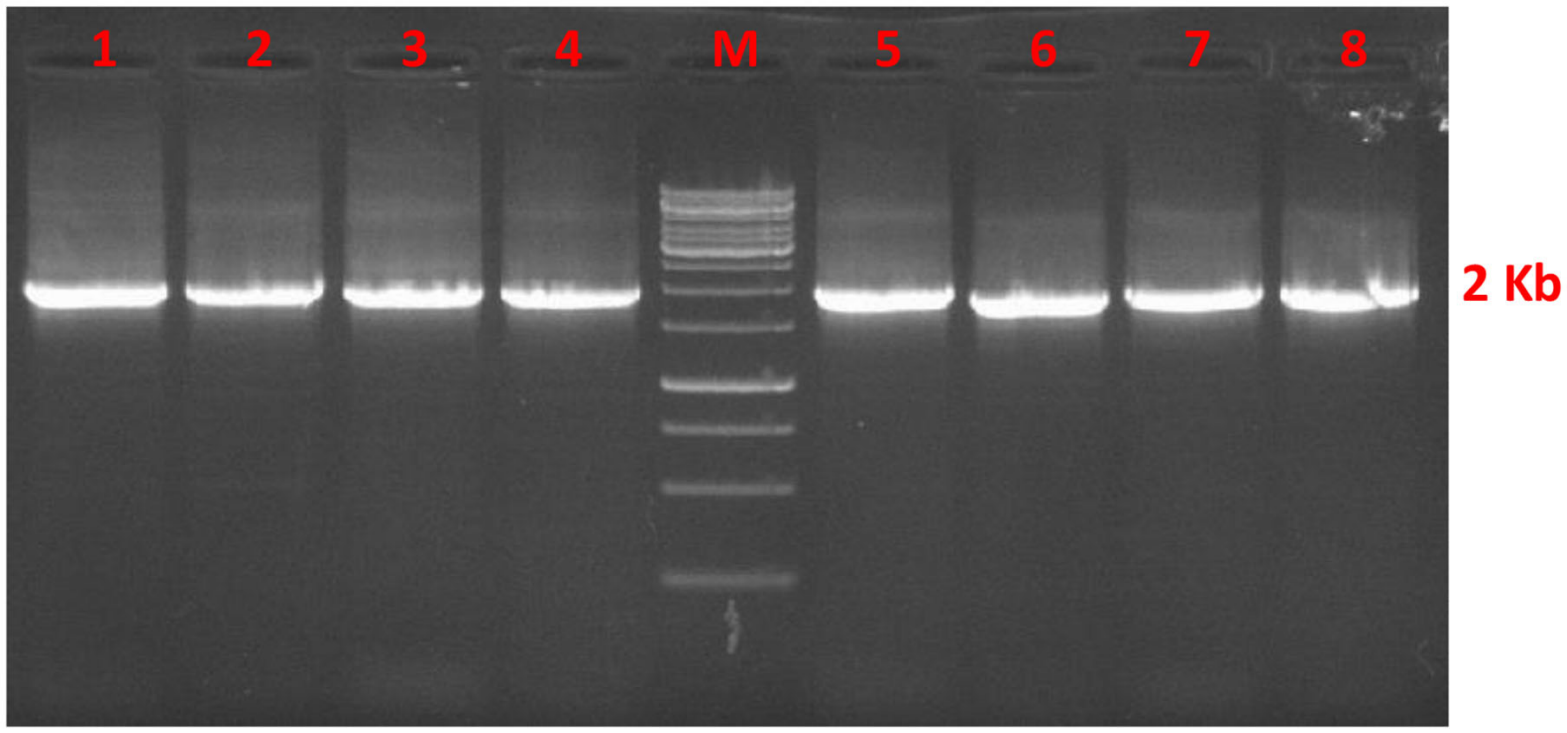
Agarose gel stained with ethidium bromide represents *AMH* gene fragment of 1728 bp from cDNA of buffalo (lane 1–4) and goat (lane 5–8) granulosa cell. Lane M is 1 Kbp DNA marker.

The *AMH* gene corresponding to 1728 base pairs was amplified from cDNA of Indian riverine buffalo and goat granulosa cells. The amplified product was checked on 1.5% agarose gel as shown in Figure 1, then followed by sequencing. The total GC content in the complete coding sequence of the *AMH* gene is 72%. The exon wise GC content were 69, 69, 73, 71, and 74%, respectively in exon I-V. The 595 amino acid residues of AMH protein consists of 24 residue signal peptides and 551 amino acid residues of the mature protein. The TGF-β domain is composed of 99 amino acids from the mature peptide. Indian riverine buffalo and goat AMH protein comprised 12 conserved cysteine residues. The highest occurring amino acids are leucine and proline accounting to 16 and 12%, respectively in the polypeptide chain. The least occurring amino acids are Met, Try, Lys, and Ile. The peptide sequences of both the animals contain two N-glycosylation sites (78-NGSR and 344-NLSD). The AMH proteins are more conserved in the C-terminal than N-terminal.

### Genetic and Syntenic Analysis of AMH

**Figure 2:**
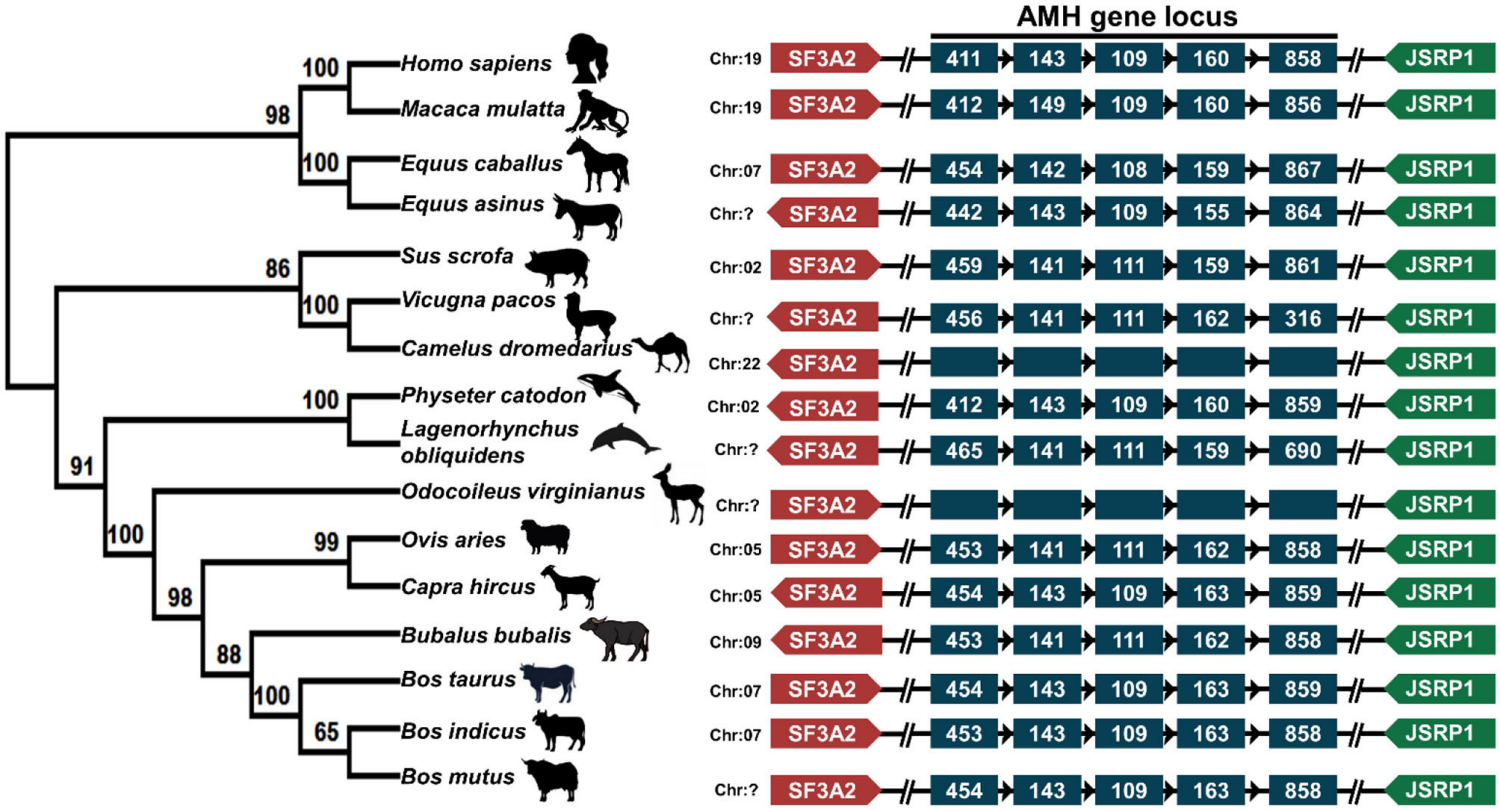
Phylogenetic and syntenic analysis of AMH: the left panel depicts the phylogenetic analysis of AMH amino acids of sixteen mammalian species performed in MegaX. The rooted phylogenetic tree is constructed from Neighbor-Joining method with bootstrap value of 1,000. The middle panel is showing the chromosomal location of *AMH* gene in respective animals. The right panel shows the syntenic analysis of the *AMH* gene of these animals. The neighboring genes *SF3A2* on the right side and J*SRP1* gene on the left side of *AMH* are represented in different colors. The arrow head represents the orientation of the genes. *AMH* gene in the middle is shown in blue color where each box representing the exon. The amino acid accession numbers for phylogenetic and syntenic analysis are: NP_000470.3 (*Homo sapiens*); NP_999475.2 (*Sus scrofa*); XP_014977898.2 (*Macaca mulatta*); NP_776315.1 (*Bos taurus*); NP_001304192.1 (*Equus caballus*); XP_014701194.1 (*Equus asinus*); XP_019820099.1 (*Bos indicus*); XP_005888453.1 (*Bos mutus*); NP_001295528.1 (*Ovis aries*); XP_020761609.1 (*Odocoileus virginianus*); XP_023990190.1 (*Physeter catodon*); QBZ68774.1 (*Bubalus bubalis*); QBZ68773 (*Capra hircus*); XP_026941471.1 (*Lagenorhynchus obliquidens*); XP_006206642.1 (*Vicugna pacos*) and XP_031293953.1 (*Camelus dromedarius*).

A phylogenetic tree of the relation of the domestic animals' AMH amino acid sequences with that of dolphin, whale and primate is presented (Figure 2). Phylogenetic analysis of AMH peptide of around 401–575 amino acid residues across the mammalian species distinctly represents two major clades, one representing Bovidae, Cetacea, Camelidae, and the other comprising the Primates and Equidae. The topology found is according to the phylogenetic grouping of cloven-hoofed, cetaceans and primates. The cloven-hoofed, ruminant vertebrates covering cattle, Indian riverine buffalo, sheep and goat are highly similar based on AMH amino acid sequence data. Cetaceans diverging from its even-toed ungulates ancestors are close to cloven-hoofed, ruminant vertebrates in phylogenetic trees. The odd-toed mammals; rhinoceros and three-toed equids are located near to each other in the phylogenetic tree and are closer to the primates. Alpaca and dromedary camels are closely related to pigs which diverged from primates and ruminant vertebrates. The molecular phylogenetic analysis of the *AMH* gene provides us with accurate descriptions of patterns of relatedness and evolutionary relationships among animal species being studied (Figure 2). The physical co-localization of *AMH* gene is in between *SF3A2* (Splicing factor 3A subunit 2) and *JSRP1* (Junctional sarcoplasmic reticulum protein 1) genes. The gene order of *SF3A2, AMH*, and *JSRP1* are highly conserved among mammalian species included in the present study. Gene encoding *AMH* and *JSRP1* in all these mammals are oriented facing each other, while gene encoding *SAF3A2* is either oriented toward *AMH* in the case of cattle, pig, monkey, horse, deer, sheep, and human or oriented opposite to AMH in the case of Indian riverine buffalo, goat, whale, dolphin, camel, and donkey (Figure 2). Around 2.1 Kbp of *AMH* consists of five exons and four introns of varying length with 72% GC content across exons and introns. The fifth exon comprises 800–900 bp making it the largest followed by exon one, four, two and three. Among mammals, the numbers of nucleotides in each exon are consistent. Higher conservation around the species is found among exon 2 and 3 regions. The matured AMH peptide consists of 575 amino acid residues with the N-terminal pro-region and the C-terminal region (TGF-β) in cattle. The signal peptide comprising of 24 amino acid residues is located in the first exon, rest part of exon one, two, three, four and some part of exon five makes N-terminal AMH and the TGF-β domain part is located at the end part of exon five (25).

### TGF-β Domain in AMH Is Conserved Across the Mammalian Species

**Figure 3:**
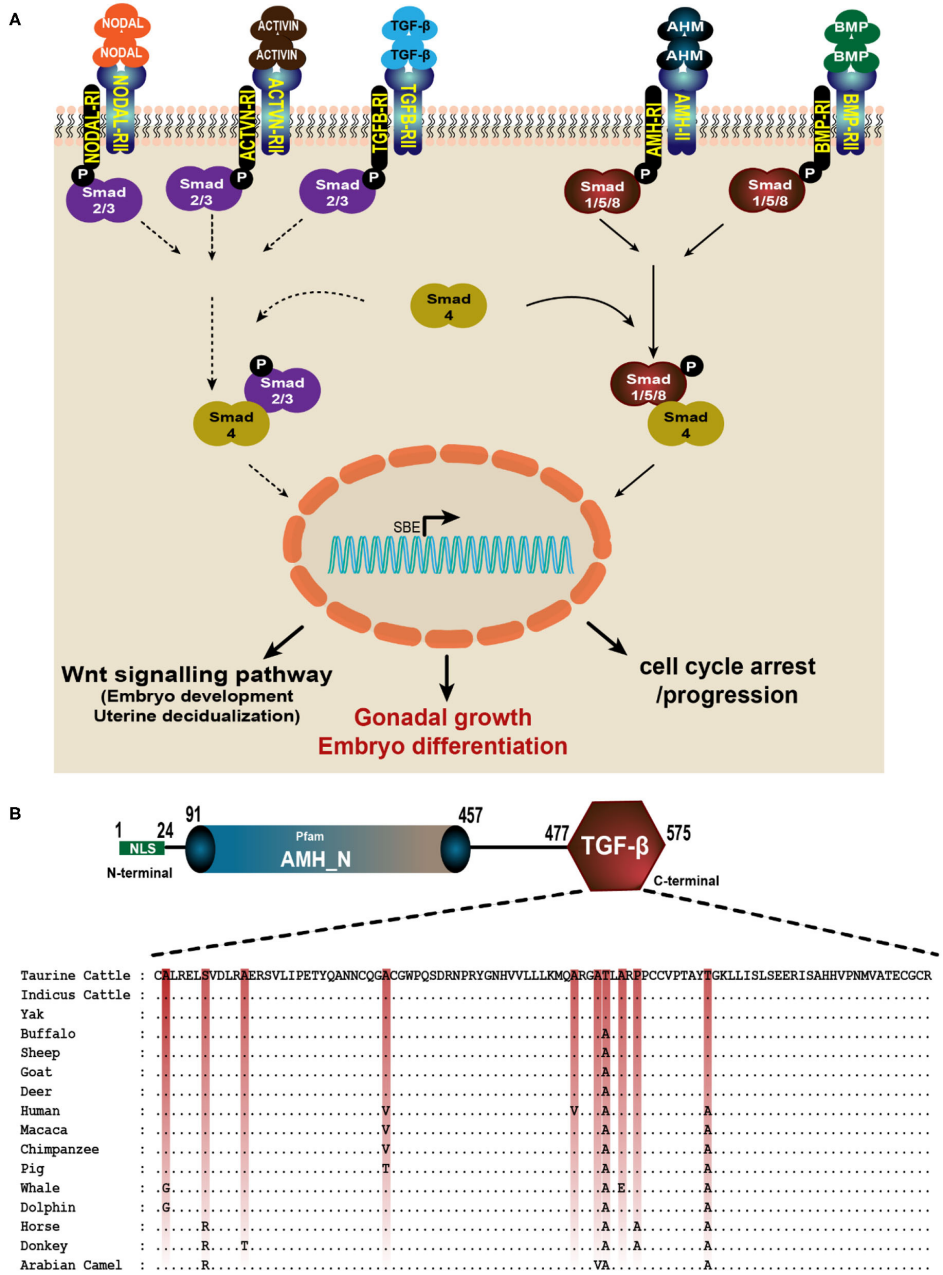
AMH is a member of TGF-β superfamily. **(A)** AMH signals through its own dedicated type II receptor (AMHRII) like other members of TGF-βs. They phosphorylate their type I receptor (AMHRI) and R-Smads (Smad1, 5, and 8). The active R-Smads then interact with co-Smad4 which translocate to the nucleus and induce various effective roles such as cell growth and differentiation, proliferation and apoptosis. **(B)** The multiple alignment of TGF-β domain in AMH gene consisting 99 amino acids is done using GeneDoc software. The amino acid accession numbers are: NP_000470.3 (Human); NP_999475.2 (Pig); XP_014977898.2 (Macaca); NP_776315.1 (Taurine Cattle); NP_001304192.1 (Horse); XP_014701194.1 (Donkey); XP_019820099.1 (Indicus Cattle); XP_005888453.1 (Yak); NP_001295528.1 (Sheep); XP_020761609.1 (Deer); XP_023990190.1 (Sperm whale); QBZ68774.1 (Buffalo); QBZ68773 (Goat); XP_026941471.1 (Dolphin) and XP_031293953.1 (Arabian Camel).

The TGF-β domain in the *AMH* gene across species is conserved with nine amino acid variations across these mammalian spices. TGF-β domain comprises 99 amino acids (from 477 to 575 in cattle, goat and sheep; 323 to 421 in yak and 462 to 560 in human), and 77 amino acids in Alpaca (from 342 to 418) amino acids. In cattle, buffalo, goat, sheep and deer the only amino acid residue located at 533 is changed from Threonine to Alanine, this change is conserved across the other species. Similarly, primates have another amino acid substitution at 505 (Alanine to Valine) and 544 (Threonine to Alanine). Cetaceans have Glycine instead of Alanine at 478, equine and camels have Arginine in place of Serine at 482. The highly conserved amino acid sequence of AMH-TGF-β domain among the mammalian type maybe for the maintained functions (Figure 3B). The positions of critical amino acids such as Methionine, Cysteine and Proline are unchanged in all the species compared for the TGF-β domain region (Figure 3B).

### Selective Pressure Analysis on *AMH* (dN/dS Ratio)

**Figure 4:**
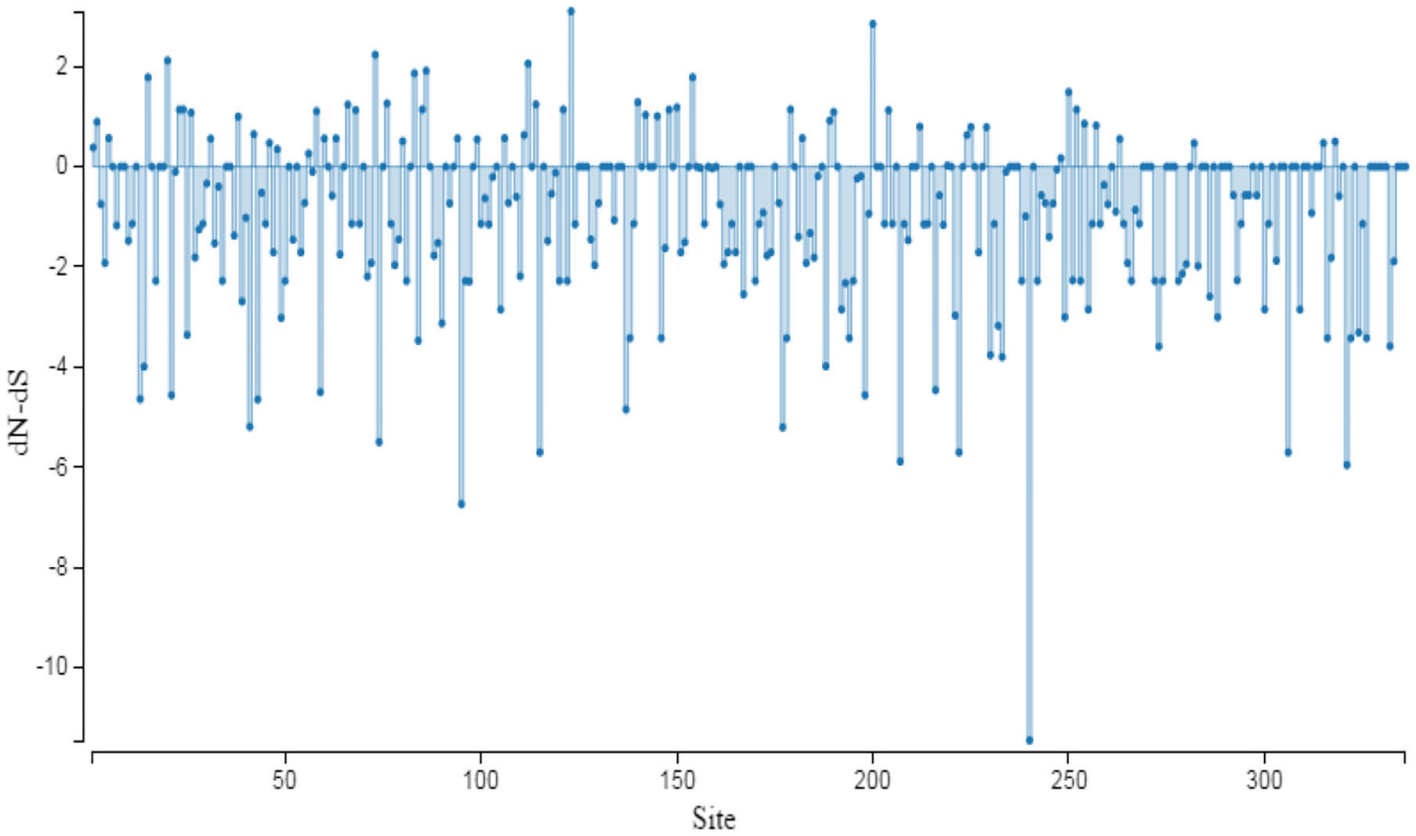
Graphic representation of dN/dS ratio for selective pressure on evolution of protein. The sites under selection are studied through SLAC method of Datamonkey server. The coding region of *AMH* gene used for analysis has the following accession numbers: NM_000479.5 (*Homo sapiens*); NM_214310.3 (*Sus scrofa*); XM_015122412 (*Macaca mulatta*); XM_016934640 (*Pan troglodytes*); NM_173890 (*Bos taurus*); NM_001317263 (*Equus caballus*); XM_014845708 (*Equus asinus*); XM_019964540 (*Bos indicus*); XM_005888391 (*Bos mutus*); NM_001308599 (*Ovis aries*); XM_020905950 (*Odocoileus virginianus*); XM_006206580 (*Vicugna pacos*); XM_024134422 (*Physeter catodon*); XM_027085670 (*Lagenorhynchus obliquidens*); XM_004441423 (*Ceratotherium simum simum*); XM_031438093 (*Camelus dromedarius*); MH479929 (*Bubalus bubalis*) and MH479928 (*Capra hircus*).

The evolutionary pressure on AMH protein is quantified by the substitution rates at non-synonymous and synonymous sites. The dN/dS ratio of this gene did not show either positive or negative selection pressure, and it therefore can be assumed that the function of AMH across species was retained (31). AMH is possibly required for the stable physiological function in reproduction of cattle, buffalo, sheep, goat, and deer. Most of the AMH codon sites are under adverse selection; the function is maintained though some sites are positively selected for a change (Figure 4).

## Discussion

AMH plays a vital role in mammalian female reproduction, especially in follicular growth and differentiation; its expression is restricted to granulosa cells of adult ovaries. The increase in follicular size increases the AMH level. The dynamic expression pattern marks an AMH to be a potential endocrine marker to the size of ovarian pool, to improve reproduction rates and for the selection of better oocyte donor (9, 13, 21, 26). In our present study, we report for the first time the complete coding sequence of *AMH* in Indian riverine buffalos and goats.

**Figure 5:**
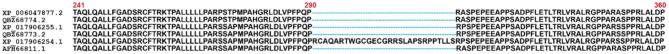
Alignment of buffalo and goat AMH proteins (QBZ68774 and QBZ68773) with reference AMH proteins (XP_006047877, XP_017906255, XP_017906254 and AFH66811). Goat AMH isoform 1 (XP_017906254.1) with additional 28 amino acids are shown.

The limited AMH sequence information in ruminant available so far may be due to technical problems in cloning and sequencing a GC-rich sequence (32, 33). Before our study, several unsuccessful attempts were made to amplify the coding sequence in Indian riverine buffalo and goat. All sequences obtained in the present study were deposited in NCBI (MH479929.2 and MH479928.2). We have compared our AMH DNA and protein sequences (MH479929.2; QBZ68774 and MH479928.2; QBZ68773) with the sequences of buffalo and goat present in the database (buffalo sequence: XP_006047877 and AFH66811, and goat sequence: XP_017906255 (isoform 2) and XP_017906254 (isoform1) obtained from whole genome shotgun sequence. Our buffalo AMH protein shared 99 and 96% identity, respectively with the database sequence XP_006047877 and AFH66811. Our goat AMH protein (QBZ68773) shared 99% identities with predicted isoform 2 (XP_017906255) and 95% identities with isoform 1 (XP_017906254). An additional twenty-eight amino acids (RCAQARTWGCGECGRRSLAPSRPPTLLS)located between position 289 and 319 of the matured protein of goat isoform 1 was not detected in any of our sequences nor in goat isoform 2 and other buffalo sequences (Figure 5). Overall, the AMH protein of buffalo and goat show a higher degree of homology in the C-terminal region compared to the N-terminal region, potentially due to the presence of the conserved TGF-β domain in the C-terminus. The presence of critical amino acids (Cys and Pro), highest and lowest occurring amino acid residue and the glycosylation sites in buffalo and goat AMH proteins were found to be similar compared to human and bovine sequences (34). The coding sequence of *AMH* in both animals contain five repeats of CCGCCC, and the same repeat sequences were also found in the promoter regions of other GC rich genes such as *c-myc, EGF* receptor and *AMH* where *sp1* transcription factor may regulate such genes containing this repeat (34). The fragile X syndrome (*FMR1*) gene exerts independent function on follicle recruitment and ovarian reserve (35). Approximately 29–30 CGG repeats on the *FMR1* gene appear reflective of usual ovarian reserve. The higher and lower CGG repeats on the *FMR1* gene denote similar risks toward premature ovarian senescence and female infertility (35). Interestingly, AMH also represents a significant association with a number of CGG repeats, a similar pattern is observed in our buffalo and goat nucleotide sequence where 46 CGG repeats are found. In the phylogenetic tree, the ruminant vertebrates covering cattle, buffalo, sheep, and goat diverging from human, primates, rhinoceros, camel, horse, and donkey may be a cause of differences in amino acid sequence composition. In Mice, AMH confined both pre-antral and early antral stages of the follicular maturation, while in primates AMH inhibitory actions are effective during antral follicle development. In non-human primate pre-antral follicles respond differently to AMH. These observations suggest the species-specific AMH behavior on follicular development and selection of dominant follicle (36, 37). In mare and women, hormonal and cyclic changes are related as they age, reproductive aging in both the species is observed with rise in FSH. The oocytes of these species are maintained in meiotic arrest for ages (38). In contrast to cattle, which only develops one or two anovulatory waves while, both mare and women develop major ovulatory as well as major and minor anovulatory waves (39). The similarities in follicle development and oocyte qualities in mare and women might be the reason that equids are closer to primates according to AMH phylogenetic studies herein. In women, AMH and AFC (Antral Follicle count) have been associated with fertility in both assisted reproduction settings as well as natural fertility in the general population (15, 40). Similarly, association between peripheral AMH concentrations and fertility of mares has been reported recently (41). The closer relationship between the equids and the primates in the phylogenetic tree might be a new finding and to our knowledge, this is the first report of associations of AMH gene between the equids and the primates. Being a member of the TGF-β family which has a vital role in cellular growth, differentiation and immunosuppression, the *AMH* gene among mammalian species has maintained its sequence integrity and their physical co-localization so, not allowing any major changes, hence are not under selection pressures.

There is a classical view of a finite primordial follicle pool in the ovaries called ovarian reserve to understand ovarian aging process better (42). During the aging process, both the number and quality of the oocytes in the ovaries decrease and reach a point beyond that, no more viable offspring may be produced and the associated cyclic endocrinological activities cease, entering the menopause in females. However, menopause like stage has not been coined in farm animal species. The ovarian reserve declines progressively with increasing chronological age within expected ranges of plasma AMH concentrations (8). Plasma AMH concentration has been demonstrated to have a high degree of correlation with ovarian antral follicle count in cattle and buffaloes (43).

The TGF-β superfamily is composed of many genes including TGF-βs, the growth and differentiation factor (GDF) subfamily, bone morphogenetic protein (BMP) subfamily, activin, inhibin, AMH and follistatin (FS) (13). Being a member of the TGF-β family, AMH exerts its biological actions by interacting with its specific anti-Müllerian hormone receptor type I (AMHRI) and anti-Müllerian hormone receptor type II (AMHRII) (44). The human *AMHRII* gene is expressed in granulosa and theca cells, and also present in other tissues such as the brain, breast and endometrium although their functional role remains elusive (11, 45). AMH signals by binding to transmembrane AMHRII and results in the phosphorylation and activation of type I receptor kinase by the constitutively active kinase domain of the type II receptor (46). The activated type I receptor then phosphorylates the cytoplasmic regulatory Smad proteins (R-Smads: 1, 5, or 8) which migrate into the nucleus, interact with co-Smad protein (Smad4) and, in concert with other transcription factors, regulate responsive genes (11). Activation of Smad4 leads to downstream signaling of various pathways like cell differentiation, apoptosis, neurogenesis, etc. (Figure 3A). The type II receptor for AMH (AMHRII) is one of five type II receptors in the TGF-β family. AMH and AMHRII are mutually specific (47). AMH involved in reproductive development, must be cleaved to bind its type II receptor (AMHRII), but dissociation of the pro-region from the mature C-terminal dimer is not required for this initial interaction (48). It was shown recently that AMH engages AMHRII at a similar interface compared to activin and BMP class ligands bind the type II receptor (AMHRII), ACVR2B; however, there are significant molecular differences at the ligand interface of these two receptors, where ACVR2B is mostly hydrophobic, and AMHRII is predominately charged. Although the location of ligand binding on the receptor is similar to ACVR2A, ACVR2B, and BMPR2; AMHRII uses unique ligand-receptor interactions to impart specificity for AMH (49).

Peripheral AMH concentrations are increasingly been used as biomarker of ovarian dysfunction in human reproduction and fertility. In patients with premature ovarian failure, AMH is undetectable or greatly reduced depending on the number of antral follicles in the ovaries (50). In contrast, AMH levels are increased in women with PCOS (18, 51). In women with PCOS, an accumulation of primary follicle was observed resulting in numbers higher than that of secondary follicles (52). However, AMH data in case of anestrus and repeat breeding has not yet been published in farm animals. Buffaloes with pubertal anestrus have been shown to have AMH deficient plasma concentration (0.53 ± 0.12 ng/ ml), while plasma AMH concentrations were found to be ≤ 2 ng/ ml in anestrus and repeat breeding cases (Unpublished data). Knowledge of *AMH* gene characterization and peripheral AMH levels in certain conditions (such as cystic ovarian syndrome, anestrus, repeat breeding condition etc.) may provide more insight into the possible cause or the effect of altered function of AMH in economic livestock animals.

## Conclusion

The entire coding region of the *AMH* gene has been sequenced for the first time from cDNA of Indian riverine buffalo and goat. Phylogenetic and syntenic analysis of *AMH* gene shows that the cloven-hoofed, ruminant vertebrates like cattle, buffalo, sheep, and goat are closely related and may be correlated with evolutionary changes in the biological process of interest; however, diverged from primates, human, camel, horse, and donkey. The *AMH* gene is positioned between *SF3A2* and *JSRP1* gene in all these mammals. The dN/dS ratio of *AMH* gene shows no positive selection pressure suggesting the similar and crucial physiological function of *AMH* gene in reproduction of these animals covering the family of Bovidae, Cetacea and Camelidae remain the same.

## Data Availability Statement

The datasets presented in this study can be found in online repositories. The names of the repository/repositories and accession number(s) can be found here: https://www.ncbi.nlm.nih.gov/, MH479929.2 and https://www.ncbi.nlm.nih.gov/, MH479928.2.

## Ethics Statement

The animal study was reviewed and approved by the Institutional Animal Ethics Committee (IACE), ICAR-NDRI, Karnal Approval no: IAEC/41/14.

## Author Contributions

DG and AV collected the samples and performed the laboratory work and analyzed the data. SD designed and supervised the research work and drafted the manuscript. AH participated in planning the research program and edited the manuscript. All the authors evaluated the manuscript.

## Conflict of Interest

The authors declare that the research was conducted in the absence of any commercial or financial relationships that could be construed as a potential conflict of interest.
